# Non-invasive Assessment of Cerebral Blood Flow and Oxygen Metabolism in Neonates during Hypothermic Cardiopulmonary Bypass: Feasibility and Clinical Implications

**DOI:** 10.1038/srep44117

**Published:** 2017-03-09

**Authors:** Silvina L. Ferradal, Koichi Yuki, Rutvi Vyas, Christopher G. Ha, Francesca Yi, Christian Stopp, David Wypij, Henry H. Cheng, Jane W. Newburger, Aditya K. Kaza, Maria A. Franceschini, Barry D. Kussman, P. Ellen Grant

**Affiliations:** 1 Fetal-Neonatal Neuroimaging & Developmental Science Center, Boston Children’s Hospital, Harvard Medical School, Boston, Massachusetts, USA; 2 Department of Anesthesiology, Perioperative & Pain Medicine, Boston Children’s Hospital, Harvard Medical School, Boston, Massachusetts, USA; 3 Department of Cardiology, Boston Children’s Hospital, Harvard Medical School, Boston, Massachusetts, USA; 4 Department of Cardiovascular Surgery, Boston Children’s Hospital, Harvard Medical School, Boston, Massachusetts, USA; 5 Athinoula A. Martinos Center for Biomedical Imaging, Massachusetts General Hospital, Harvard Medical School, Charlestown, Massachusetts, USA

## Abstract

The neonatal brain is extremely vulnerable to injury during periods of hypoxia and/or ischemia. Risk of brain injury is increased during neonatal cardiac surgery, where pre-existing hemodynamic instability and metabolic abnormalities are combined with long periods of low cerebral blood flow and/or circulatory arrest. Our understanding of events associated with cerebral hypoxia-ischemia during cardiopulmonary bypass (CPB) remains limited, largely due to inadequate tools to quantify cerebral oxygen delivery and consumption non-invasively and in real-time. This pilot study aims to evaluate cerebral blood flow (CBF) and oxygen metabolism (CMRO_2_) intraoperatively in neonates by combining two novel non-invasive optical techniques: frequency-domain near-infrared spectroscopy (FD-NIRS) and diffuse correlation spectroscopy (DCS). CBF and CMRO_2_ were quantified before, during and after deep hypothermic cardiopulmonary bypass (CPB) in nine neonates. Our results show significantly decreased CBF and CMRO_2_ during hypothermic CPB. More interestingly, a change of coupling between both variables is observed during deep hypothermic CPB in all subjects. Our results are consistent with previous studies using invasive techniques, supporting the concept of FD-NIRS/DCS as a promising technology to monitor cerebral physiology in neonates providing the potential for individual optimization of surgical management.

Given its critical functions, the brain is the ultimate organ whose outcome must be optimized in any operating room or intensive care setting. All surgical and medical management strategies must be designed to prevent or at least minimize brain injury. Neonates undergoing cardiac surgery with cardiopulmonary bypass (CPB) are particularly vulnerable to hypoxic-ischemic brain injury^1^,^2^,^3^. Since CPB requires periods of reduced systemic flow and/or circulatory arrest, cerebral hypothermia is routinely used with a primary goal to reduce cerebral oxygen consumption, such that the risk of hypoxic-ischemic brain injury is minimized. Laboratory and human studies have shown that hypothermia produces a significant neuroprotective reduction in cerebral oxygen consumption^4^,^5^. Despite the fact that hypothermia and new surgical techniques have led to a significant improvement in survival rates, infants undergoing cardiac surgery continue to show abnormal long term neurodevelopmental outcomes^6^. To date there is no information to demonstrate that current neuromonitoring methods prevent brain injury and improve neurodevelopmental outcomes^7^,^8^,^9^,^10^,^11^. Our understanding of perioperative cerebral hypoxia-ischemia in neonates undergoing complex cardiac surgery remains limited, primarily due to the lack of adequate tools to quantify cerebral oxygen delivery and consumption non-invasively, in real-time, and at the bedside.

Seminal work by Greeley and colleagues showed that cerebral metabolism is exponentially related to temperature during hypothermic CPB in neonates, infants and children^12^. They also showed that long periods of deep hypothermic circulatory arrest (DHCA) alter cerebral metabolism and blood flow after the arrest period despite adequate hypothermic suppression of metabolism^13^. Antegrade regional cerebral perfusion (RCP) has been proposed as a better perfusion technique to avoid or minimize duration of circulatory arrest and maximize oxygen delivery to the brain^14^. However, clinical outcomes available so far do not provide strong evidence of the superiority of RCP over DHCA^15^. This has led to a wide variation in practice and the fundamental question of whether it is possible to optimize cerebral oxygen delivery to match cerebral oxygen consumption in individual patients. Yet, optimizing cerebral oxygen delivery first requires a technique for monitoring cerebral metabolism with minimal risk for the patient.

The techniques used by Greeley *et al*. 25 years ago to measure cerebral metabolism are invasive and/or require exposure to radioactive Xenon^12^,^13^. Greeley’s methods are also technically challenging to perform in the operating room, and are not feasible in routine clinical practice. As a result, these experiments have not been repeated. Currently, intraoperative brain health is assessed by monitoring oxygenation with continuous-wave near infrared spectroscopy (CW-NIRS). CW-NIRS is limited to work as a trend monitor, as it cannot measure absolute concentrations of oxy- (HbO_2_) and deoxy-hemoglobin (HbR). Consequently, it cannot provide absolute cerebral oxygen saturation (SO_2_) but only relative changes (rSO_2_). This lack of quantification limits the estimation of normative values and undermines comparisons between normal and at-risk neonates. Recently, the advanced non-invasive optical techniques of frequency-domain near-infrared spectroscopy (FD-NIRS) and diffuse correlation spectroscopy (DCS) have demonstrated the ability to not only measure absolute cerebral oxygen saturation but also provide quantitative measures of cerebral blood volume (CBV) and a quantitative index of cerebral perfusion (CBF_i_), enabling a quantitative index of cerebral oxygen consumption (CMRO_2i_) to be calculated^16^,^17^,^18^,^19^. These advanced optical technologies have the potential to provide similar quantitative information regarding brain health as the invasive methods of Greeley with the advantages of lower risk to the patient and the same ease of use as current commercial NIRS devices.

This pilot study aims to evaluate the ability of FD-NIRS/DCS to quantify intraoperative cerebral blood flow and oxygen metabolism non-invasively in neonates. Because of the extreme physiologic manipulations induced by changes in perfusion and temperature (CPB, deep hypothermia, and regional cerebral perfusion), neonatal cardiac surgery provides an excellent opportunity to evaluate SO_2_, CBF_i_ and CMRO_2i_ intraoperatively. If clinical translation of these technologies becomes feasible, they hold great promise in monitoring cerebral health in real-time, providing the potential for individual optimization of cerebral oxygen delivery to match cerebral metabolic demands.

## Results

**Figure 1:**
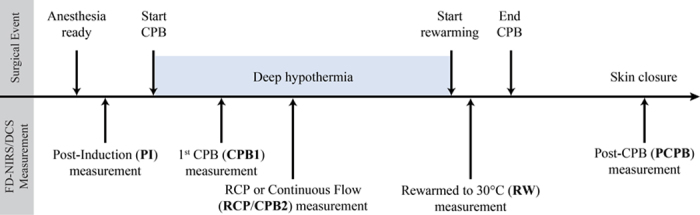
Timeline of intraoperative measurements.

Cardiac diagnosis and CPB parameters for each patient are summarized in Table 1. Nasopharyngeal temperature was maintained less than 24 °C in all patients during hypothermic CPB. Brief DHCA was utilized in all subjects. Seven patients received RCP following brief DHCA. Two patients had continuous flow CPB instead. A timeline of the measurements is shown in Fig. 1 with measurements made at 5 time points (see Methods for a detailed description of the measurement protocol).

Table 2 summarizes the physiologic parameters at each time point, including pH and PaCO_2_ values uncorrected for body temperature. Hematocrit (Hct, %) was maintained in the range (27–37) % during CPB.

### Cerebral hemodynamics during neonatal cardiac surgery measured by FD-NIRS/DCS

**Figure 2:**
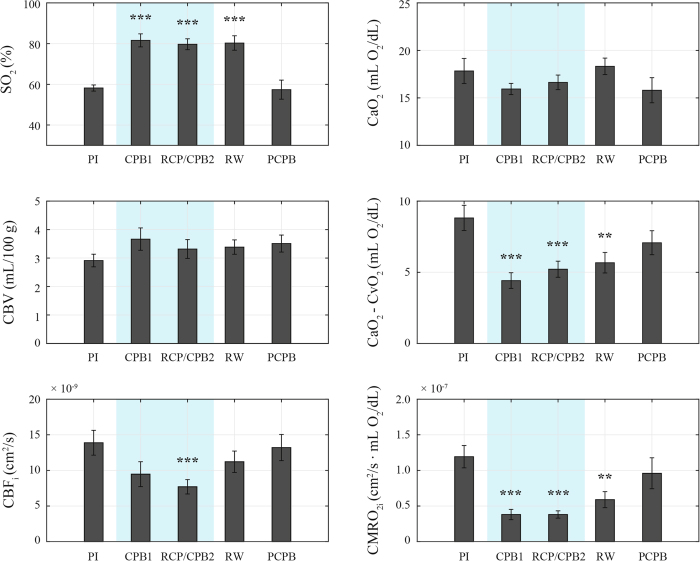
Hemodynamic measurements before, during and after CPB across all subjects. Error bars represent SEM values. Shaded regions indicate measurements taken during deep hypothermia. Statistical significance relative to post-induction (PI) is noted.

Significant changes in cerebral oxygenation and blood flow were found at the different stages of surgery (Fig. 2). In particular, SO_2_, (CaO_2_ − CvO_2_), CBF_i_ and CMRO_2i_ showed statistically significant changes with respect to baseline (PI stage) during deep hypothermia (shaded regions). After weaning from CPB, SO_2_, (CaO_2_ − CvO_2_), CBF_i_ and CMRO_2_ were not significantly different from baseline. No significant changes were registered in CBV and CaO_2_.

The variability of our measurements was assessed by computing a coefficient of variation (COV). The average COV for SO_2_ and CBF_i_ measurements (consisting of 3 repetitions) was 2.4% (SEM = 0.4%) and 10.7% (SEM = 0.5%), respectively.

### CBF_i_/CMRO_2i_ coupling

**Figure 3:**
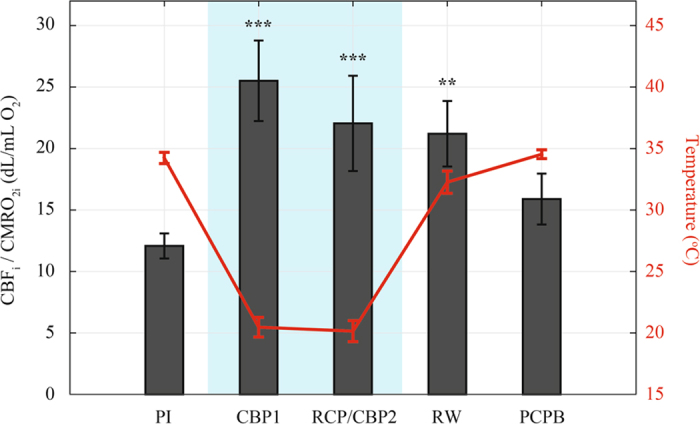
Coupling between cerebral blood flow and oxygen consumption during cardiac surgery. Mean NP temperature is overlaid at each stage. Note the considerable increase in the CBFi/CMRO_2i_ ratio, or decrease in oxygen extraction, during deep hypothermia relative to post-induction (PI). Error bars represent SEM values. Shaded regions indicate measurements taken during deep hypothermia.

The CBF_i_/CMRO_2i_ ratio provides an indicator of the coupling between both parameters at different stages (Fig. 3). During deep hypothermia, the CBF_i_/CMRO_2i_ ratio showed a significant increase with respect to baseline (*p* < 0.005), indicating cerebral blood flow in excess of cerebral demand. This ratio decreased during rewarming (but remained significantly higher from the baseline) and returned to baseline levels after weaning from CPB (PCPB stage).

### Relationship between temperature and CMRO_2i_

**Figure 4:**
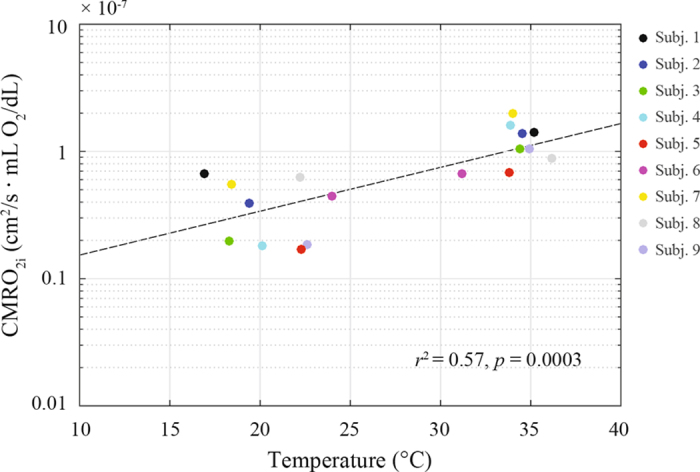
Effect of temperature on CMRO_2i_ during CPB.

Figure 4 shows the temperature dependency of CMRO_2i_ during CPB in a temperature range of 16 °C to 35 °C. The relationship can be described by an exponential function: CMRO_2i_ = 0.066 e^0.08T^ (*r*^2^ = 0.57, *p* = 0.0003). In order to rule out the effect of circulatory arrest, only values corresponding to baseline (PI) and early CPB (CPB1) (before circulatory arrest or RCP) were considered in the linear regression between temperature and the logarithm of CMRO_2i_.

### Neurological complications

The intraoperative and postoperative course was uneventful in 7/9 subjects with no neurological signs or symptoms triggering a postoperative neurological consult or clinical neuroimaging. One neonate (subject ID: 8) had an unexpected decrease in CBF_i_ in the post cardiopulmonary bypass phase (lowest observed but within one standard deviation of the group mean). This neonate required a neurological consult due to a complicated cardiac surgery, extended bypass and concern for intraoperative hypoperfusion and hypoxic ischemic injury. A post-operative research MRI obtained on this subject 13 days after surgery showed multiple foci of white matter injury of unclear etiology as a preoperative MRI was not obtained. However intraoperative white matter injury due to hypoperfusion cannot be excluded. This neonate was discharged with no focal neurological deficit but is too young for neurocognitive testing. Another neonate (subject ID: 4) required ECMO immediately after surgery. This neonate also required a neurological consult due to poor cardiac output and need for ECMO. Care was eventually withdrawn due to uncorrectable complex congenital heart disease. This neonate had high CBF_i_ post induction but was too unstable for post bypass measures.

## Discussion

In this pilot study, we used a custom-made FD-NIRS/DCS system to quantify cerebral blood flow and oxygen metabolism during neonatal cardiac surgery. While several studies have employed these techniques to monitor neonatal cardiac surgical patients preoperatively and/or postoperatively^18^,^19^,^20^,^21^, this is the first report of intraoperative CBF_i_ and CMRO_2i_ measurements in neonates undergoing hypothermic CPB. The patterns observed in our measurements are in agreement with previous results obtained with invasive techniques in a similar population^12^, suggesting that FD-NIRS/DCS is a promising technology to monitor individual cerebral oxygen metabolism and hemodynamics during complex neonatal cardiac surgical procedures.

While neurological complications in adult cardiac surgery are most commonly embolic in nature, those in pediatric populations are more frequently related to hypoperfusion and hypoxia, supporting the importance of understanding the balance between cerebral oxygen delivery (provided by CBF) and consumption. Greeley *et al*. assessed these variables by using Xenon-clearance methods and jugular bulb oximetry^12^,^13^. In studies conducted in neonates and children, CMRO_2_ decreased exponentially while CBF decreased linearly during hypothermic CPB, leading to a relative excess of blood flow, a situation referred to as “luxury perfusion”. Our findings using non-invasive optical methods are consistent with these reports. Specifically, we showed a significant decrease ( >60%) of CMRO_2i_ during hypothermic CPB relative to baseline (Fig. 2), which accompanied an exponential decay with temperature (Fig. 4). While CBF_i_ also decreased, it exhibited a more moderate reduction (31%) during hypothermia with respect to baseline values (Fig. 2). The variability of our SO_2_ and CBF_i_ measurements (COV_SO2_ = 2.4% and COV_CBFi_ = 10.7%) is below the physiological changes observed at the different stages, suggesting that our observations are driven by physiological fluctuations.

Our results also show a change of coupling between cerebral blood flow and oxygen consumption (Fig. 3). This is illustrated by the CBF_i_/CMRO_2i_ ratio, or the inverse of oxygen extraction, that exhibits a two fold increase of the average ratio during deep hypothermic bypass relative to baseline. Flow/metabolism coupling is a complex process influenced by many parameters (temperature, PaCO_2_, pH, etc.). As body temperature decreases and pH increases with hypothermia, in the pH stat strategy CO_2_ is added to the blood during CPB in order to maintain a pH of 7.4 at the patient’s actual body temperature. Thus, the increase in CBF_i_ relative to CMRO_2i_ during deep hypothermia could be partially explained by the counteracting effect between temperature and PaCO_2_, as it has been shown that CO_2_ is a strong cerebral vasodilator during hypothermic CPB and has the potential to disrupt cerebral autoregulation^22^,^23^. The mechanism and consequences of the change of coupling are still incompletely understood. The luxury perfusion is thought to provide an element of cerebral protection and hence positively influence neurodevelopmental outcome^5^. However, the influence of hypothermia and its interaction with other variables during CPB is complex. Hypothermia increases the O_2_ affinity of hemoglobin so that the fraction of CMRO_2_ dependent on dissolved O_2_ increases at deep hypothermia^24^. Dissolved O_2_ can theoretically meet the brain O_2_ requirements if CBF is adequate. An important question for future studies will be to improve our understanding of the implications of hypothermia itself and its relationship to physiological variables such as CBF and CMRO_2_ and neurodevelopmental outcomes. A potential approach for studying this question is by studying groups of patients with a similar diagnosis undergoing CPB with deep (<22 °C) versus mild-moderate (28–35 °C) hypothermia.

Commercial CW-NIRS systems have been previously used to measure cerebral oxygenation in neonates undergoing cardiac surgery^25^,^26^. As discussed above, CW-NIRS systems only provide relative measurements of cerebral oxygenation (rSO_2_) and are prone to inaccuracies in the high and low ends of the scale^27^,^28^.Our preliminary comparisons between SO_2_ and rSO_2_ (as measured by a commercial system Fore-Sight, CAS Medical Systems Inc., Branford, CT) show that while both measurements seem to follow a similar trend, rSO_2_ overestimates cerebral oxygen saturation, particularly in the lower ranges (Supplementary Figure 1). A more systematic study with a larger sample needs to be performed in order to explain this bias.

SO_2_ alone does not offer a measure of cerebral perfusion. As discussed by Elwell and colleagues, SO_2_ belies the complexity of the multiple processes determining cerebral oxygen delivery and consumption^29^. Cerebral blood flow velocity measured by TCD is a reliable surrogate of cerebral blood flow in large arteries provided that the diameter of the vessel remains constant, an assumption that might not be met during CPB. Moreover, as the TCD signal depends on the angle of insonation and is thus operator dependent, it is technically challenging to obtain consistent measurements, hindering accurate real-time assessment of cerebral blood flow. DCS is complementary to TCD as it measures cerebral blood flow in the microvasculature by measuring light intensity fluctuations caused by the movement of red blood cells^30^,^31^. While absolute CBF_i_ does not have units of cerebral blood flow, it has shown a strong correlation with cerebral blood flow measured by bolus tracking techniques using time-domain NIRS in a neonatal pig model^32^ as well as in preterm neonates^33^, and with cerebral blood flow measured by phase-encoded velocity mapping using magnetic resonance imaging in neonates^34^. Since both DCS and FD-NIRS are sensitive to microvasculature underneath the optical sensor, they offer better estimates of CMRO_2_ than by combining SO_2_ NIRS with other modalities like TCD which measure blood velocity in large vessels and may not accurately reflect cortical perfusion at the site of the probe.

This pilot study has several limitations. Because of routine use of a commercial cerebral oximeter and limited space on the forehead, we only performed measurements overlying the right frontal cortex. Future studies will include additional locations such as parietal and temporal regions in order to account for potential regional variability of cerebral blood flow and metabolism.

While FD-NIRS and DCS have the ability to perform continuous measurements of SO_2_ and CBF_i,_ we only considered a number of short measurements to simplify the probe design and avoid confounding effects such as motion and instrument drift. Future implementations will consider the design of ergonomic probes that can be attached to the infant’s head throughout the entire surgery and newer systems with improved stability, thus providing continuous measurements of perfusion and oxygenation.

The goal of this pilot study was to test the ability to track major metabolic changes due to extreme physiologic manipulations. Despite the small sample size (N = 9), we were able to obtain statistically significant changes at different stages of the surgery that are consistent with previous reports from larger studies. However, larger datasets will be required to answer subtler questions such as the effect of perfusion techniques (DHCA versus RCP) on cerebral blood flow and oxygenation, or metabolic variability associated with specific cardiac defects.

In summary, this pilot study demonstrated the ability to non-invasively track the extreme cerebral metabolic changes during neonatal cardiac surgery. Our results are consistent with previous reports from invasive studies and support the idea of FD-NIRS/DCS as a promising technology to monitor individual cerebral physiology in neonatal patients.

## Methods

The study protocol was reviewed and approved by the Boston Children’s Hospital Committee on Clinical Investigation. The study method was designed and carried out in accordance with the BCH Committee on Clinical Investigation requirements and the regulations that govern human subjects’ research.

### Subjects, anesthetic and CPB management

Nine neonates (median gestational age: 39 weeks; median weight: 3.1 kg) admitted to the Cardiac Intensive Care Unit at Boston Children’s Hospital were enrolled in the study. Parents who agreed to participate were asked to read and sign an informed consent form as approved by the BCH Committee on Clinical Investigation. Only neonates undergoing cardiac surgery for congenital heart disease within 30 days of birth were considered eligible. Infants with a known or suspected syndrome, craniofacial abnormality, sepsis, metabolic disorder, brain malformation, or brain mass lesion were excluded from the study.

All patients underwent palliative or reparative cardiac surgery within the first week of life (median post-natal age at surgery: 4 days). Anesthesia consisted of high-dose opioid (fentanyl ~100 mcg/kg) and muscle relaxant, supplemented with isoflurane and/or midazolam as tolerated. CPB was non-pulsatile with a target hematocrit of 30% at initiation of bypass. Ice was placed around the head and moved aside during NIRS measurements. Temperature was monitored in the nasopharynx as a reflection of brain temperature per our routine practice. In addition, arterial and venous return temperatures during CPB were recorded. A pH-stat strategy (blood gas management with temperature correction) was used during core cooling, low-flow hypothermic bypass, and regional cerebral perfusion (RCP). Mean duration of cooling was 21 minutes (Fig. 1). CPB parameters are summarized in Table 1.

### Study protocol

A total of 5 measurements were performed on each patient (Fig. 1). First, a baseline measurement (post-induction; PI) was made under stable conditions following induction of anesthesia, endotracheal intubation, and placement of arterial and venous lines, shortly before skin incision. The second measurement (CPB1) was performed at the end of core cooling, *i.e.*, when the patient reached the cooling target temperature (mean ± SD: 21 ± 4 minutes after initiation of CPB). The third measurement was made after 15 minutes of RCP (RCP; n = 7; 16 ± 5 minutes after start of RCP) or after an hour of continuous flow bypass (CPB2; n = 2; 57 ± 10 minutes after onset of CPB). The timing of the third measurement for both the RCP patients and continuous flow patients was similar (~60 minutes after onset of CPB). A fourth measurement (RW) was done when the patient was rewarmed to 30 °C (nasopharyngeal temperature) and prior to cardiac ejection. Given the fast dynamics of this stage, some measurements were performed within a broader range of temperatures (27 °C–35 °C). The fifth measurement (PCPB) was performed when the patient was stable and ready to leave the OR. In all stages, the probe was placed on the right side of the infant’s forehead. A commercial cerebral oximeter was placed on the left side of the forehead as part of routine clinical use.

### Instrumentation and optical data analysis

The FD-NIRS system (OxiplexTS, ISS Inc., Champaign, IL, USA) has two banks of laser diode sources operating at 8 wavelengths (670–830) nm and two photomultiplier tube (PMT) detectors. The laser sources are modulated at 110 MHz, and the PMT detectors operate with a modulation of 110 MHz + 5 kHz for heterodyne detection at 5 kHz. The DCS system has a long coherence length laser source operating at 785 nm and four photon-counting avalanche photodiode (APD) detectors. An eight-channel correlator is used to convert the detected light at each DCS detector channel into temporal intensity autocorrelation functions over a delay time range of (2 10^−7^–0.5) seconds.

A custom-made probe arranges FD-NIRS and DCS sources and detectors in a single row, allowing for FD-NIRS measurements at four source-detector (SD) distances (1.5, 2.0, 2.5, and 3.0 cm) and DCS measurements at a single SD distance (2.0 cm). Fiber bundles are used to couple FD-NIRS sources and detectors. The DCS laser is coupled to a multimode optical fiber and diffused at the fiber tip to provide power levels that comply with the ANSI standard. Each of the four DCS detectors is coupled to a single mode optical fiber. The four DCS detection single-mode fibers are placed at the same SD distance in order to increase signal-to-noise ratio of the detected DCS signal.

For FD-NIRS, absorption (μ_a_) and scattering (μ_s_’) of the sampled tissue were estimated from the amplitude attenuation and phase shift measured at each wavelength and SD distance using the multi-distance frequency-domain method^35^. Measurements taken on a phantom block with known optical properties were used to estimate coupling coefficients between tissue and optical fibers. Absolute oxygenated (HbO_2_) and deoxygenated (HbR) hemoglobin were derived from the wavelength-dependent absorption coefficients using hemoglobin extinction coefficients reported in the literature^36^ and assuming a water concentration of 75%^37^. Cerebral oxygen saturation was defined as 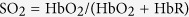.

For DCS, each measured intensity autocorrelation function was fit to obtain a cerebral blood flow index (CBF_i_) using the analytical semi-infinite solution to the correlation diffusion equation for a homogenous medium^30^,^38^. Individual FD-NIRS derived absorption and scattering coefficients at 785 nm were used in the fitting of CBF_i_ at each measured time point (Supplementary Figure 2). Note that the DCS technique provides a measure of microvascular perfusion by quantifying intensity fluctuations of multiply scattered light due to the movement of red blood cells inside the sampled tissue. CBF_i_, measured in cm^2^/sec, has been validated as a measure of regional cerebral blood flow^32^,^33^,^34^.

The optical probe was sanitized and inserted into a single use plastic cover. The FD-NIRS/DCS system was kept outside the sterile surgical site. FD-NIRS and DCS measurements were repeated three times per surgery time point on the infant’s right forehead. For each repetition, the probe was repositioned in a slightly different area to account for local inhomogeneities such as hair and superficial large vessels. FD-NIRS/DCS acquisition time was set to 20 seconds and the three repeated measurements were averaged together to obtain a single value at each time point.

We assessed reproducibility of the SO_2_ and CBF_i_ measurements by calculating a coefficient of variation (COV = standard deviation/mean) for each set of three measurements for each every subject included in the study.

### Measurements of brain metabolism and blood values

An index of the cerebral metabolic rate of oxygen (CMRO_2i_, cm^2^/s · mL O_2_/dL) was calculated using Fick’s principle:


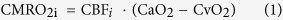


where CaO_2_ is the oxygen content of arterial blood and CvO_2_ is the oxygen content of mixed venous blood. The oxygen content CxO_2_ (mL O_2_/dL), with x = a or v denoting arterial or venous blood, was calculated according to the equation:


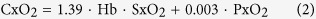


where Hb (g/dL) is the hemoglobin concentration in blood, SxO_2_ (%) is the arterial or venous oxygen saturation and PxO_2_ (mmHg) is the partial pressure of oxygen in arterial blood or mixed venous blood.Venous oxygen saturation, SvO_2_, was derived from SO_2_ measured with FD-NIRS and SaO_2_ obtained from the arterial blood sample using the following equation:


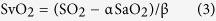


where α + β = 1, and β = 0.75 is the percent contribution of the venous compartment to the SO_2_ measurement^39^.

Cerebral blood volume (CBV, mL/100 g) was calculated as


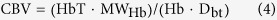


where HbT (μMol) is the total hemoglobin concentration, MW_Hb_ (65,400 g/Mol) is the molecular weight of hemoglobin, and D_bt_ (1.05 g/ml) is the brain tissue density. Finally, oxygen extraction was calculated as (CaO_2_ − CvO_2_).

### Statistics

All values are given as mean ± standard error of the mean (SEM) unless stated otherwise. Changes between baseline (PI) and other time points were tested using a Wilcoxon two-sided signed-rank test. The dependency of CMRO_2i_ on brain temperature was tested by linear regression analysis. Pearson correlations were used to examine the relationship of CPB flow rate with CBF_i_ and mean arterial pressure (MAP). A *p*-value < 0.05 was considered to be statistically significant. In tables and figures, *indicates *p* < 0.05, **indicates *p* < 0.005, and ***indicates *p* < 0.0005.

## Additional Information

**How to cite this article:** Ferradal, S. L. *et al*. Non-invasive Assessment of Cerebral Blood Flow and Oxygen Metabolism in Neonates during Hypothermic Cardiopulmonary Bypass: Feasibility and Clinical Implications. *Sci. Rep.* **7**, 44117; doi: 10.1038/srep44117 (2017).

**Publisher's note:** Springer Nature remains neutral with regard to jurisdictional claims in published maps and institutional affiliations.

## Supplementary Material

Supplementary Information

## Figures and Tables

**Table 1:** Cardiac diagnosis and cardiopulmonary bypass parameters.

ID	Diagnosis	Flow rate @ CPB1 (mL/kg/min)	Flow rate @ RCP/CPB2 (mL/kg/min)	Flow rate @ RW (mL/kg/min)	Longest DHCA duration (min)	Longest RCP duration (min)	Nadir temp. on CPB (°C)
1	HLHS	172	39	207	10	87	15.4
2	HLHS	173	24	138	3	57	21.1
3	d-TGA	165	76	156	11	—	18.1
4	d-TGA with aortic atresia, VSD	119	29	174	4	54	18.7
5	Type B IAA with VSD	173	43	178	10	65	21.1
6	d-TGA	111	104	141	8	—	23.8
7	HLHS	139	40	212	8	72	17.5
8	d-TGA, ASD, VSD, and hypoplastic arch	119	28	169	20	73	21.3
9	HLHS	126	39	161	8	44	19.8

HLHS: hypoplastic left heart syndrome; d-TGA: dextro-transposition of great arteries; VSD: ventricular septal defect; IAA: interrupted aortic arch; ASD: aortic septal defect; CPB: cardiopulmonary bypass; DHCA: deep hypothermic circulatory arrest; RCP: regional cerebral perfusion.

**Table 2:** Summary of physiologic parameters (mean ± SEM) at each time point and significance of change relative to PI.

	PI	CPB1	RCP/CPB2	RW	PCPB
Temperature (°C)	34.2 ± 0.5	20.5 ± 0.8 ***	20.2 ± 0.9 ***	31.3 ± 0.9	34.5 ± 0.4
Hct (%)	42.5 ± 2.6	30.3 ± 1.2 **	32.0 ± 1.6 **	36.6 ± 1.9	39.7 ± 2.3
Hb (g/dL)	13.8 ± 0.9	9.8 ± 0.4 **	10.3 ± 0.5 **	11.9 ± 0.6	12.9 ± 0.8
SaO_2_ (%)	91.6 ± 1.7	99.9 ± 0.1 ***	99.8 ± 0.1 ***	99.7 ± 0.1 ***	86.3 ± 6.8
pH	7.36 ± 0.02	7.32 ± 0.02 ***	7.35 ± 0.01 ***	7.43 ± 0.02	7.34 ± 0.04
PaCO_2_ (mmHg)	43.8 ± 2.7	52.4 ± 7.4	47.8 ± 7.0	38.0 ± 1.5 *	46.1 ± 3.5
PaO_2_ (mmHg)	57.5 ± 4.1	608.9 ± 7.7 ***	589.4 ± 11.9 ***	600.8 ± 6.1 ***	144.0 ± 45.9

*Indicates *p* < 0.05, **indicates *p* < 0.005, and *** indicates *p* < 0.0005.
