# Supplementary material for: Non-invasive Assessment of Cerebral Blood Flow and Oxygen Metabolism in Neonates during Hypothermic Cardiopulmonary Bypass: Feasibility and Clinical Implications
Source: Sci Rep. 2017 Mar 9;7:44117. doi: 10.1038/srep44117 (PMC5343476; doi:10.1038/srep44117)
Supplement: Supplementary Information [file srep44117-s1.pdf]

Supplemental Material for

Non-invasive Assessment of Cerebral Blood Flow and Oxygen Metabolism in Neonates during Hypothermic Cardiopulmonary Bypass: Feasibility and Clinical Implications

Silvina L. Ferradal[1]*, Koichi Yuki[2]*, Rutvi Vyas[1], Christopher G. Ha[1], Francesca Yi[1], Christian Stopp[3], David Wypij[3], Henry H. Cheng[3], Jane W. Newburger[3], Aditya K. Kaza[4], Maria A. Franceschini[5], Barry D. Kussman[2#] and P. Ellen Grant[1#]

[1]Fetal-Neonatal Neuroimaging & Developmental Science Center, Boston Children's Hospital, Harvard Medical School, Boston, Massachusetts, USA

[2]Department of Anesthesiology, Perioperative & Pain Medicine, Boston Children's Hospital, Harvard Medical School, Boston, Massachusetts, USA

[3]Department of Cardiology, Boston Children's Hospital, Harvard Medical School, Boston, Massachusetts, USA

[4]Department of Cardiovascular Surgery, Boston Children's Hospital, Harvard Medical School, Boston, Massachusetts, USA

[5]Athinoula A. Martinos Center for Biomedical Imaging, Massachusetts General Hospital, Harvard Medical School, Charlestown, Massachusetts, USA

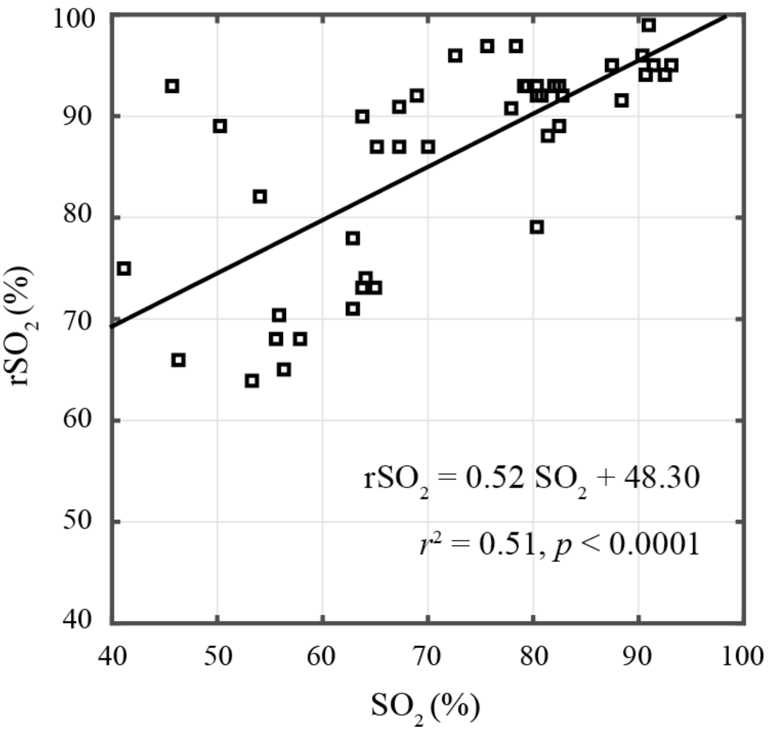

Supplementary Figure 1: Correlation between cerebral oxygen saturation (rSO_2) measured by a commercial CW-NIRS oximeter (Fore-Sight, CAS Medical Systems, Inc., Branford, CT) and oxygen saturation (SO_2) measured by FD-NIRS across all subjects and time points.

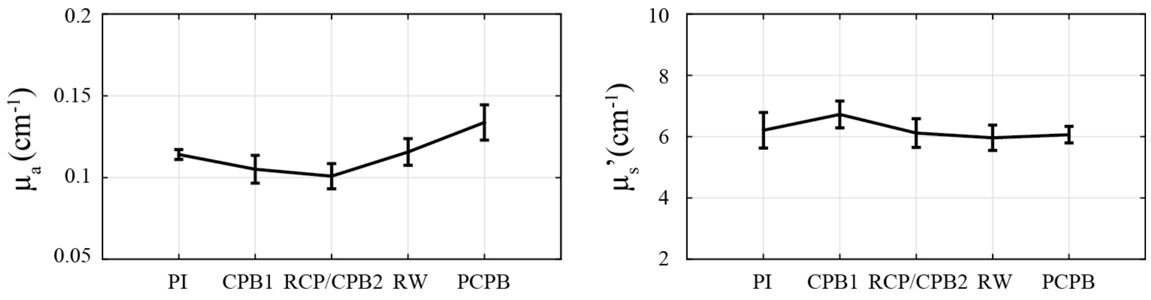

Supplementary Figure 2: Average optical properties (absorption and scattering) obtained at each time point.
